# Malignant glioma subset from actuate 1801: Phase I/II study of 9-ING-41, GSK-3β inhibitor, monotherapy or combined with chemotherapy for refractory malignancies

**DOI:** 10.1093/noajnl/vdac012

**Published:** 2022-02-07

**Authors:** Yazmin Odia, Ludimila Cavalcante, Howard Safran, Steven Francis Powell, Pamela N Munster, Wen Wee Ma, Benedito A Carneiro, Bruno R Bastos, Stacy Mikrut, William Mikrut, Francis J Giles, Solmaz Sahebjam

**Affiliations:** 1 Department of Neuro-Oncology, Miami Cancer Institute, Baptist Health South Florida, Miami, Florida, USA; 2 Actuate Therapeutics, Fort Worth, Texas, USA; 3 Department of Hematology Oncology, Cancer Center at Brown University, Lifespan Cancer Institute, Providence, Rhode Island, USA; 4 Department of Medical Oncology, Sanford Health, Sioux Falls, South Dakota, USA; 5 Department of Hematology Oncology, University of California San Francisco, San Francisco, California, USA; 6 Department of Medical Oncology, Mayo Clinic, Rochester, Minnesota, USA; 7 Vantage Data Designs, Austin, Texas, USA; 8 Department of Neuro-Oncology, Moffitt Cancer Center & Research Institute, University of South Florida, Tampa, Florida, USA

**Keywords:** gliomas, GSK-3β, 9-ING-41, lomustine, phase I trial

## Abstract

**Background:**

GSK3β serine/threonine kinase regulates metabolism and glycogen biosynthesis. GSK3β overexpression promotes progression and resistance through NF-κB and p53 apoptotic pathways. GSK3β inhibits immunomodulation by downregulating PD-L1 and LAG-3 checkpoints and increasing NK and T-cell tumor killing. 9-ING-41, a small-molecule, selective GSK3β inhibitor, showed preclinical activity in chemo-resistant PDX glioblastoma models, including enhanced lomustine antitumor effect.

**Methods:**

Refractory malignancies (*n* = 162) were treated with 9-ING-41 monotherapy (*n* = 65) or combined with 8 cytotoxic regimens after prior exposure (NCT03678883). Recurrent gliomas (*n* = 18) were treated with 9-ING-41 IV TIW q21day cycles at 3.3, 5, 9.3, 15 mg/kg, as monotherapy or combined with lomustine 30 mg/m² PO weekly q84day cycles. Primary objective was safety.

**Results:**

RP2D of 15 mg/kg IV TIW was confirmed across all 9 regimens, no accentuated chemotherapy toxicity noted. Glioma subtypes included: 13 glioblastoma, 2 anaplastic astrocytomas, 1 anaplastic oligodendroglioma, 1 astrocytoma. Median age 52 (30–69) years; 6 female, 12 male; median ECOG 1 (0–2); median recurrences 3 (1–6). All received upfront radiation/temozolomide (18/18), plus salvage nitrosoureas (15/18), bevacizumab (8/18), TTFields (6/18), or immunotherapy (4/18). IDH/mutation(3/18); 1p19q/codeletion(1/18); MGMT/methylated(1/18). Four received 9-ING-41 monotherapy, 14 concurrent with lomustine. No severe toxicities were attributed to 9-ING-41, only mild vision changes (9/18, 50%), or infusion reactions (4/18, 22%). Lomustine-related toxicities: G3/4 thrombocytopenia (3/14, 21%), G1/2 fatigue (4/14, 28%). Median days on therapy was 55 (4–305); 1 partial response (>50%) was noted. Median OS was 5.5 (95% CI: 2.8–11.4) months and PFS-6 was 16.7%.

**Conclusion:**

9-ING-41 plus/minus lomustine is safe and warrants further study in glioma patients.

**Key Points**

- FIH dose-escalation trial of 9-ING-41, GSK-3β inhibitor, plus/minus lomustine.
- No grade 3–4 9-ING-41-related nor accentuated grade 3–4 lomustine toxicities noted.
- Preliminary OS and PFS were promising and warrant further study.

**Importance of the Study**

This study reports the safety and preliminary efficacy signal of the first-in-human study of 9-ING-41, a small-molecule potent selective GSK-3β inhibitor, as monotherapy or in combination with lomustine in adults with recurrent gliomas. GSK-3β, a serine/threonine kinase, is a key regulator of metabolism and glycogen biosynthesis. GSK-3β aberrant overexpression promotes tumor progression and chemotherapy resistance through NF-κB and p53-mediated apoptotic pathways. 9-ING-41 showed preclinical antitumor activity against several tumor types, including chemo-resistant PDX models of glioblastoma, where 9-ING-41 enhanced the antitumor effect of lomustine. 9-ING-41 proved safe as single agent and in combination with lomustine in adult gliomas. The combination of weekly low dose lomustine plus 9-ING-41 warrants further study in patients with glioblastoma.

Based on the Central Brain Tumor Registry of the United States (CBTRUS), gliomas are the most frequent adult primary brain tumors with an incidence of 6.03 per 100 000 adults per year. Glioblastoma, WHO grade IV glioma, is the most frequent adult primary malignant brain tumor accounting for 14.5% of all primary brain tumors.^1^ Malignant gliomas are the second leading cause of cancer mortality in adults under 35 years of age.^2^ Despite advances in imaging, anesthesia and surgical techniques, the prognosis of malignant gliomas treated by surgical resection alone is dismal with a median survival of 4–6 months.^3,4,5^ Radiotherapy remains the most effective treatment, extending median survival to 8–9 months.^6,7,8^ Temozolomide therapy extends median survival to 15 months for glioblastomas and 2–5 years for anaplastic gliomas.^9,10^ Tumor treating fields (TTFs) are low intensity, moderate frequency, alternating electrical fields that added to adjuvant temozolomide prolonged median survival for glioblastomas to 20 months.^11^ The 5-year survival rate for glioblastoma remains at <10%.^12^ Cytotoxic agents or other antineoplastic therapies have limited efficacy in recurrent disease with an expected overall survival of 3 months for glioblastoma.^5^ Malignant gliomas remain a significant unmet clinical need with dismal survival and limited effective treatment options. There is an urgent need for innovative, safe, and effective therapies for these uniformly fatal neoplasms.

Glycogen Synthase Kinase-3 (GSK-3) is a serine/threonine kinase initially described as a key regulator of metabolism, specifically glycogen biosynthesis.^13^ It has a role in diverse disease processes including cancer, immune disorders, metabolic disorders, and neurological disorders through modulation of a large number of substrates. GSK-3 has two ubiquitously expressed and highly conserved isoforms, GSK-3α and GSK-3β, with both shared and distinct substrates and functional effects.^14–20^

GSK-3β is particularly important in tumor progression and oncogene modulation (including beta-catenin, cyclin D1, and c-Myc), cell cycle regulators (e.g., p27Kip1), and mediators of epithelial-mesenchymal transition (e.g., zinc finger protein SNAI1, Snail).^15,21–23^ Aberrant overexpression of GSK-3β has been shown to promote tumor growth and chemotherapy resistance in various solid tumors including colon, ovarian and pancreatic cancers and glioblastoma through differential effects on the pro-survival nuclear factor kappa-light-chain-enhancer of activated B cells (NF-κB) and c-Myc pathways as well on tumor necrosis factor-related apoptosis-inducing ligand (TRAIL) and p53-mediated apoptotic mechanisms.^24–29^

NF-κB is a transcription factor which is constitutively active in tumor cells and promotes anti-apoptotic molecule expression; its activation is particularly important in chemo- and radio-resistant cancer cells.^30^ GSK-3β is a positive regulator of NF-κB and a pro-oncogene, therefore, inhibiting GSK-3β could overcome NF-kB-mediated chemo-resistance in human cancers.

In GBM, molecular analysis of brain tumor biopsies has identified elevated expression of NF-κB and its target genes compared to normal brain tissue.^31^ Constitutive activation of NF-κB has been reported in human GBM tumors and found to be important in promoting tumor invasion and resistance to alkylating agents.^32^ GSK3 inhibition induces glioma cell death through c-MYC, NF-κB, and glucose regulation. GSK3 inhibition was accompanied by downregulation of several NF-κB regulated pro- survival genes including IL8, IER3, and BIRC2 as assessed by microarray gene expression analysis and TaqMan RT-PCR. In addition, the inhibition of GSK3 activity results in c-MYC activation leading to the induction of Bax, Bim, DR4/DR5, and TRAIL expression and subsequent cytotoxicity in glioma models. Other tool GSK3 inhibitors and TRAIL act synergistically in glioma cell cytotoxicity both *in vitro* and *in vivo*. Targeting components of NF-κB signaling represents a therapeutic strategy to overcome GBM chemo-resistance.

9-ING-41 is a first-in-class, intravenously (IV) administered, maleimide-based small molecule and a potent selective GSK-3β inhibitor with significant preclinical single agent antitumor activity that involves G0-G1 and G2-M phase arrest and induction of apoptosis (Figure 1).^23,33,34^ The putative antitumor mode of action of 9-ING-41 is through downregulation of NF-κB and decrease in the expression NF-κB target genes cyclin D1, Bcl-2, anti-apoptotic protein (XIAP), and B-cell lymphoma-extra-large (Bcl-XL), leading to inhibition of tumor growth in multiple solid tumor and lymphoma cell lines, as well as patient derived xenograft (PDX) models.^27,35,36^ The mechanism for apoptosis is through caspase-3 cleavage.^37^ In the synthesis and selection of 9-ING-41, the compound was chosen from a panel of several GSK3-B inhibitors due to its optimal CNS penetration.^38^

**Figure 1.**
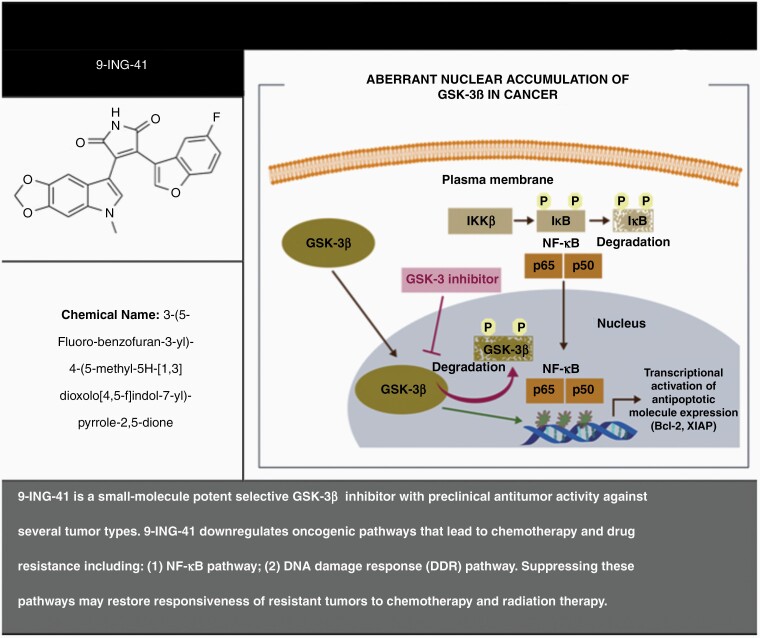
Mechanism of Action of GSK-3ß and 9-ING-41 (GSK-3ß inhibitor). 9-ING-41 is a small-molecule potent selective GSK-3β inhibitor with preclinical antitumor activity against several tumor types. 9-ING-41 downregulates oncogenic pathways that lead to chemotherapy and drug resistance including: (1) NF-κB pathway; (2) DNA damage response (DDR) pathway. Suppressing these pathways may restore responsiveness of resistant tumors to chemotherapy and radiation therapy.

In both chemo- and radio-resistant orthotopic PDX models of GBM, the combination of 9-ING-41 and CCNU demonstrated significant regression of established intracranial tumors and histologically confirmed cures.^34^ A chemosensitive GBM model demonstrated durable and long responses in mice treated with lomustine + 9-ING-41, with complete tumor regression and increase in mouse weight and health. Median survival was 142 days vs. 85 without 9-ING-41. Complete remission was also noted in chemo-resistant glioblastoma orthotopic tumors in mice, with complete survival after treatment with CCNU + 9-ING-41 and total regression of intracranial GBM6 PDX tumors with recovery of mouse brain structures. Based on the preclinical data reported by Ugolkov *et al* (2017),^35^ the regimen chosen required interaction between 9-ING-41 and the chemotherapy more frequently than the standard dosing of lomustine dosed every 6 weeks. The weekly lomustine regimen chosen was developed by the MDACC group with documented clinical data as detailed by CA Koller *et al* (1994) in a phase I trial of weekly lomustine in patients with advanced cancer. The safety profile was preferable and allowed for frequent concurrent dosing with the twice weekly 9-ING-41 infusions. These results provided a strong rationale for advancing 9-ING-41 into clinical development for treating glioma patients.

## Methods

### Study Design

**Figure 2.**
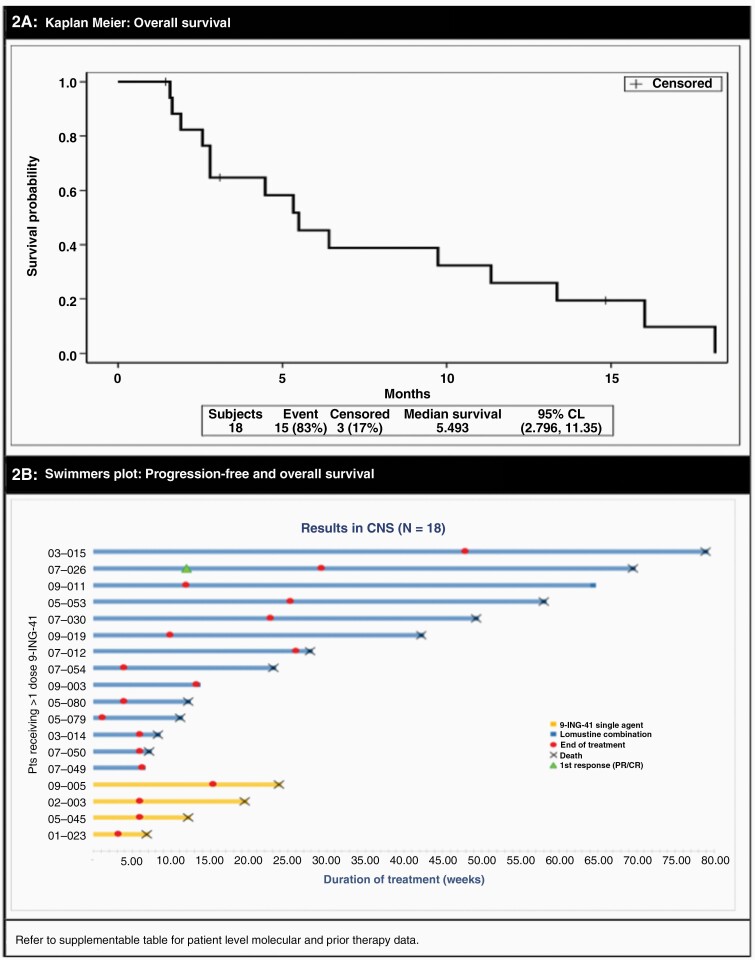
Survival Statistics for the Glioma Subset Kaplan-Meier Overall Survival curve (A) and Swimmer Plot (B) of Progression-Free and Overall Survival for the glioma cohort. A supplemental table includes individual molecular and prior therapy data for patients as represented in the swimmer plot.

This was a first-in-human trial (NCT03678883) of 9-ING-41 as monotherapy or combined with 8 cytotoxic regimens after prior treatment with the same chemotherapy for refractory malignancies. The primary objective was to evaluate the safety and tolerability, describe any dose-limiting toxicity (DLT), determine the maximum tolerated dose (MTD) or highest protocol-defined doses (in the absence of exceeding the MTD) and the recommended phase II study dose (RP2D) for 9-ING-41 as monotherapy (Study Part 1) and in combination with chemotherapies (Study Part 2) in patients with relapsed or refractory malignancies. Secondary analyses included progression-free survival, overall survival, clinical benefit rate, and duration of response. For the glioma cohort, secondary endpoints included response by RANO (Response assessment in neuro-oncology criteria) (Figure 2).^38,39^

### Ethics

An appropriate institutional review board or ethics committee approved the project. Written informed consent was obtained from the subjects or legally authorized representative.

### Toxicity Assessment

Safety was assessed throughout the study including by recording and monitoring Adverse events (AEs) based on the CTCAE v4.03. Standard monitoring included vital signs (blood pressure, pulse, respiratory rate, body temperature), physical examination findings, serum chemistry and hematology laboratory values, urinalysis, ECG, and concomitant medication usage. For the purpose of dose escalation, a dose-limiting toxicity was defined as any prolonged or clinically significant grade 3-4 adverse event newly occurring in the first 21 days of the first cycle of treatment, unless there is a clear alternative explanation (e.g., related to underlying disease/progression). Grade ≥3 infusion reactions or other allergic reaction or anaphylaxis was not being considered a DLTs.

### Study Treatment and Assessments

Enrollment into the monotherapy arm of the study (*n* = 67) followed a traditional 3 + 3 design for 8 different dose levels of 9-ING-41 (1.0, 2.0, 3.3, 5.0, 7.0, 9.3, 12.4, and 15 mg/kg) given IV twice a week in 21-day cycles. In the 8 chemotherapy combination arms (*n* = 171), enrollment started after 2 dose levels cleared in the monotherapy arm and followed a 3 + 3 dose escalation design in tandem with monotherapy escalation, with 6 dose levels of 9-ING-41 tested (3.3, 5.0, 7.0, 9.3, 12.4, and 15 mg/kg). Intra-patient and inter-patient dose escalation of 9-ING-41 was allowed if subsequent dose levels cleared and patients continued on study. Patients were treated until progression, unacceptable toxicity, or until no longer deriving benefit from therapy.

Main inclusion criteria for the overall study included: refractory malignancy, age ≥18 years, ECOG PS 0–3, prior treatment with same chemotherapy regimen to be administered in the combination arms, and stable CNS disease for 14 days prior to starting therapy on study. Patients with GBM and other CNS tumors were required to have measurable disease, defined as a clearly enhancing tumor with at least two perpendicular diameters at entry ≥1 cm. Patients with gliomas also needed histologic confirmation with unequivocal progression after chemoradiotherapy with or without antiangiogenic treatment at least 3 months after the end of radiotherapy.

The subset of patients with recurrent gliomas (*n* = 18) was treated with 9-ING-41 monotherapy IV twice weekly in 21-day cycles at different dose levels or with 9-ING-41 given IV twice weekly combined with lomustine 30 mg/m² PO once weekly in 84-day (12 week) cycles. Response assessment was based on MRIs required every 12 weeks, though investigators often opted for MRIs every 6 weeks per local practice, using the response assessment in neuro-oncology (RANO) criteria. Patients on the lomustine arm were eligible if failed prior therapy with a nitrosurea.

## Results

### Study Population

#### Demographics.

There were 6 female and 12 male patients. The median age at study entry was 52 (30–69) years. The median ECOG at study entry was 1 (0–2). The histologies included 13 glioblastoma, 2 anaplastic astrocytomas, 1 anaplastic oligodendroglioma, 1 diffuse astrocytoma. All patients had received first-line radiation and temozolomide (18/18) prior to study enrollment. The median number of recurrences and lines of therapy for recurrent disease were 3 (1–6). Prior therapy for recurrences included nitrosoureas (15/18), bevacizumab (8/18), TTFields (6/18), and immune checkpoint inhibitor (4/18) (Table 1).

**Table 1.** Study Design Plus Summary Statistics for the Glioma/Lomustine Subset

Part 1: Enrolling Specific Patient Cohorts @ RP2D of Single Agent 9-ING-41							
Part 2: Define RP2D of 9-ING-41 in Combination with Standard Chemotherapy Chosen by Investigator Based on Diagnosis and Prior Treatment: 9-ING-41 Plus							
Gemcitabine 1250 mg/m^2^ IV Days 1/8 of a 21-day Cycle	Doxorubicin 75 mg/m^2^ IV Day 1 of a 21-day Cycle	Carboplatin AUC 6 IV Day 1 of a 21-day Cycle	Irinotecan 350 mg/m^2^ IV Day 1 of a 21-day Cycle	Lomustine 30 mg/m^2^ PO as Single Dose Weekly in an 84-Day Cycle	Nab-Paclitaxel 125 mg/m^2^ IV and Gemcitabine 1000 mg/m^2^ IV Days 1, 8, and 15 of a 28-day Cycle	Paclitaxel 175 mg/m^2^ IV and Carboplatin IV AUC 6 on Day 1 of a 21-day Cycle	Pemetrexed 500 mg/m^2^ IV Carboplatin IV AUC 5 on Day 1 of a 21-day Cycle
Demographics							Descriptive Statistic
Enrolled (*n*)							18
Age (median, min–max)							52.5 (30–70)
Gender (female, %)							6 (33.3%)
Race/ethnicity (*n*, %)							18 (100%) White, 2 (11.2%) Hispanic
ECOG 1 (*n*, %)							13 (72.2%)
Prior therapies (median, min–max)							3 (1–5)
Histology and genetics					Descriptive statistic		
Histology^a^ (*n* = 18)					Glioblastoma		14 (77.8%)
					Malignant IDH-mutantAstrocytoma		2 (11.1%)
					Astrocytoma		1 (5.6%)
					Anaplastic Oligodendroglioma		1 (5.6%)
Genomics					IDH mutation		3/18 (16.7%)
					1p19q codeletion		1/18 (5.6%)
					ATRX mutation		2/11 (18%)
					MGMT promoter methylation		1/18 (5.5%)
					EGFR amplification		7/11 (64%)
					EGFR v3 mutation		4/11 (36%)
					TERT promoter mutation		8/11 (73%)
					ASXL1 mutation		1/11 (9%)
					CDKN2A/B deletion		2/11 (18%)
					NF1 rearrangement		1/11 (9%)
					PALB2 mutation		1/11 (9%)
					PTEN loss		4/11 (36%)
					RB1 loss		1/11 (9%)
					TP53 mutation		3/11 (27%)
Outcomes							Descriptive Statistic
Evaluable for response (*n*, %)							18 (100%)
Partial response (*n*, %)							1 (5.6%)
6-month PFS (%)							3 (16.7%)
Median PFS (months, range)							1.9 (0.3–11.1)
Median OS (months, 95% CI)							5.5 (95% CI: 2.8–11.4)

Abbreviations: KPS, Karnofsky performance status; OS, Overall Survival; PFS, progression-free survival.

^a^Histology based on 2021 WHO criteria included 2 “molecular” glioblastomas (histology favoring anaplastic astrocytoma) and WHO grade IV IDH-mutant astrocytoma (no long glioblastoma based on molecular profile).

### Genetic Profile of the Glioma Subset

Glioma subtypes included 1 anaplastic oligodendroglioma (IDH-mutant, 1p/19q codeleted) and 2 other IDH-mutant astrocytomas, 1 IDH wild-type anaplastic astrocytoma, plus 14 glioblastomas with wild-type or unknown IDH status. Key genomic alterations for the 18 patients plus additional 11 NGS reports included: MGMT promoter methylated in 1/18 (5.5%); EGFR amplification in 7/11 (64%) and EGFR v3 mutation in 4/11 (36%); TERT promoter mutation in 8/11 (73%); ATRX loss in 2/11 (18%); TP53 mutated in 3/11 (27%), PTEN loss in 4/11 (36%); CDKN2A deletion in 2/11 (18%); NF1 rearrangement in 1/11 (9%); RB1 loss in 1/11 (9%); ASXL1 mutation 1/11 (9%); and PALB2 mutation 1/11 (9%).

### Adverse Events

A 9-ING-41 RP2D of 15 mg/kg IV twice weekly was confirmed across all 9 regimens (monotherapy and 8 different chemotherapy combinations). Dose escalation halted at 15 mg/kg due to volume of administration cap at 2L per dose, determined to be the maximum clinically feasible dose. No DLTs or 9-ING-41 attributed SAEs were observed in the monotherapy arm. In the combination arms, one SAE was observed in a patient with grade 3 transient vision change unable to perform ADLs for the duration of the event. Refer to Table 2 for complete list of drug related toxicities.

**Table 2.** Toxicity of 9-ING-41 Monotherapy and in Combination with Lomustine

CTCAEv4.03 Term	Grade 1–2 (*n*, %)		Grade 3–4 (*n*, %)		All Grade (*n*, %)	
9-ING-41 IV twice weekly monotherapy						
Any TEAE	**12**	**66.0%**	0	0.0%	**12**	**66.0%**
Visual disturbances	9	50.0%	0	0.0%	**9**	**50.0%**
IV site injury	3	16.7%	0	0.0%	**3**	**16.7%**
Infusion Reaction	3	16.7%	0	0.0%	**3**	**16.7%**
Fatigue	2	11.1%	0	0.0%	**2**	**11.1%**
Amylase Increased	1	5.6%	0	0.0%	**1**	**5.6%**
Lipase increased	1	5.6%	0	0.0%	**1**	**5.6%**
9-ING-41 IV twice weekly in combination with lomustine PO once weekly						
Any TEAE	**5**	**27.8%**	3	16.7%	**4**	**44.4%**
Anemia (hemoglobin)	0	0.0%	1	5.6%	**1**	**5.6%**
Leukopenia (WBC)	0	0.0%	1	5.6%	**1**	**5.6%**
Lymphopenia (ALC)	1	5.6%	0	0.0%	**1**	**5.6%**
Neutropenia (ANC)	1	5.6%	0	0.0%	**1**	**5.6%**
Thrombocytopenia (platelets)	2	11.1%	2	11.1%	**4**	**44.4%**
Purpura	1	5.6%	0	0.0%	**1**	**5.6%**
Nausea	1	5.6%	0	0.0%	**1**	**5.6%**
Stomatitis	1	5.6%	0	0.0%	**1**	**5.6%**
Fatigue	6	33.3%	0	0.0%	**6**	**33.3%**
iv site injury	1	5.6%	0	0.0%	**1**	**5.6%**
Decrease Appetite	1	5.6%	0	0.0%	**1**	**5.6%**
Epistaxis	1	5.6%	0	0.0%	**1**	**5.6%**

No Grade 3–4 Treatment-Emergent Adverse Event (TEAE) were reported for 9-ING-41 monotherapy.

In the subset of glioma patients (*n* = 18), there was no accentuation of chemotherapy-related toxicity noted. Four subjects with recurrent gliomas received single agent 9-ING-41, while 14 were treated with 9-ING-41 IV twice weekly plus lomustine 30 mg/m^2^ orally once weekly. Those patients receiving lomustine were required to have prior exposure to a nitrosourea. No SAEs or grade 3/4 adverse events were attributed to 9-ING-41. In this subset of patients, 9-ING-41-related toxicities included grades 1/2 transient vision changes (9/18, 50%) and infusion reactions (4/18, 22%). Transient vision changes consisted of patients reporting lights brighter and skin tones darker, effects lasted up to several hours and were completely reversible without any end-organ damage. This side effect was considered a potential sign of target engagement and a drug class effect, due to the known prevalence of GSK-3B in the synaptic layers of the retina and photoreceptor cells.^40^ Side effects from lomustine included grades 3/4 thrombocytopenia (3/14, 21%) and grade 1/2 fatigue (4/14, 28%) as expected. No attributable grade 5 events were observed in this cohort, nor in the overall study.

### Clinical Endpoints

**Figure 3.**
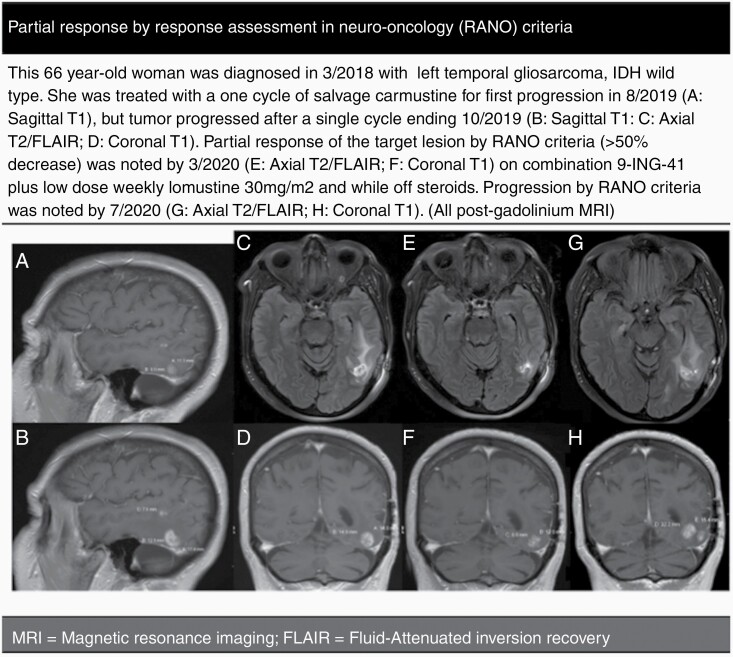
Partial response by response assessment in neuro-oncology (RANO) criteria. This 66-year-old woman was diagnosed in 3/2018 with a left temporal gliosarcoma, IDH wild-type. She was treated with a one cycle of salvage carmustine for first progression in 8/2019 (A: Sagittal T1), but tumor progressed after a single cycle ending 10/2019 (B: Sagittal T1; C: Axial T2/FLAIR; D: Coronal T1). Partial response of the target lesion by RANO criteria (>50% decrease) was noted by 3/2020 (E: Axial T2/FLAIR; F: Coronal T1) on combination 9-ING-41 plus low dose weekly lomustine 30 mg/m^2^ and while off steroids. Progression by RANO criteria was noted by 7/2020 (G: Axial T2/FLAIR; H: Coronal T1). (All postgadolinium MRI).

**Figure 4.**
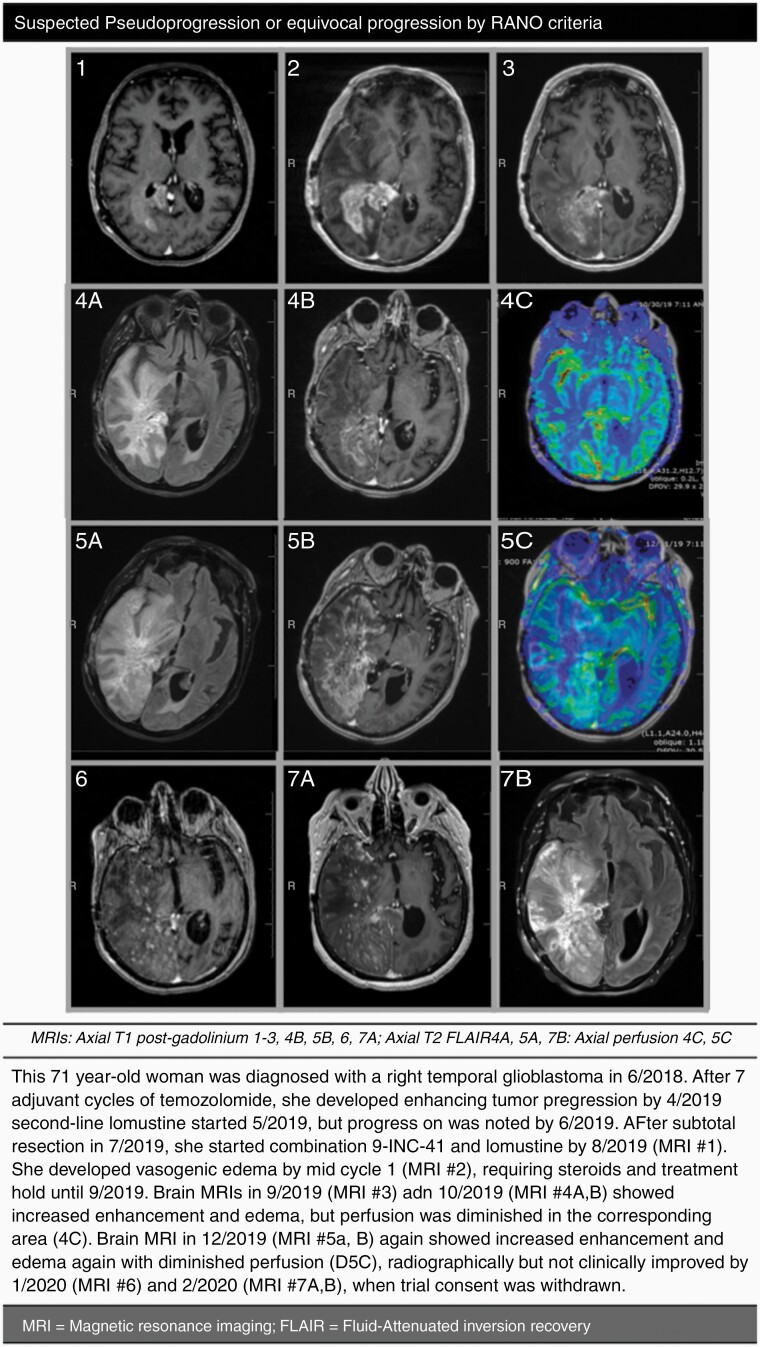
Suspected pseudo-progression or equivocal progression by RANO criteria. This 71-year-old woman was diagnosed with a right temporal glioblastoma in 6/2018. After 7 adjuvant cycles of temozolomide, she developed enhancing tumor progression by 4/2019. Second-line lomustine started 5/2019, but progression was noted by 6/2019. After subtotal resection in 7/2019, she started combination 9-ING-41 and lomustine by 8/2019 (MRI #1). She developed vasogenic edema by mid cycle 1 (MRI #2), requiring steroids and treatment hold until 9/2019. Brain MRIs in 9/2019 (MRI #3) and 10/2019 (MRI #4A,B) showed increased enhancement and edema, but perfusion was diminished in the corresponding area (4C). Brain MRI in 12/2019 (MRI #5A,B) again showed increased enhancement and edema again with diminished perfusion (5C), radiographically but not clinically improved by 1/2020 (MRI #6) and 2/2020 (MRI #7A,B), when trial consent was withdrawn.

In the glioma subgroup, median duration on therapy was 55 (4–305) days or <1 cycle, and 4 out of 18 patients (22%) had stable disease for 20 weeks (or ~2 cycles) or longer. The median progression-free survival (PFS) was 1.9 (0.3–11.1) months, median overall survival (OS) of 5.5 (95% CI: 2.8–11.4), and PFS at 6 months of 16.7% (Table 1; Figure 2). Best overall response observed was 1 partial response after 2 cycles of 9-ING-41 and lomustine (Figure 3), noted in an IDH wild-type gliosarcoma that had progressed on carmustine prior to trial enrollment. Pseudo-progression, or increased enhancement with low cerebral perfusion, was suspected in a patient with glioblastoma (Figure 4). Symptoms often improved with treatment hold and/or steroids, with stabilization if not improved enhancement on subsequent MRIs.

## Discussion

In this phase I trial, no significant toxicities were attributed to 9-ING-41, only mild and transient vision changes (50%) or infusion reactions (22%). Lomustine-related toxicities, including moderate-to-severe thrombocytopenia (21%) and mild fatigue (28%) were comparable to rates and severity reported in monotherapy trials.

Most gliomas enrolled had at least 3 prior lines of therapy. The observed survival compares favorably with historical controls in the ≥2nd line GBM setting, after failure of temozolomide.^41–44^ Additionally, in this ≥3^rd^ line recurrent population, all patients received first-line radiation and temozolomide (18/18), and prior therapies for recurrences included nitrosoureas (15/18), bevacizumab (8/18), TTFields (6/18), and immune checkpoint inhibitors (4/18).

A patient developed symptomatic intra-cerebral edema following infusion with 9-ING-41, requiring courses of steroids and treatment holds of the investigational agent until symptom resolution and/or radiographic improvement. This suspected “pseudoprogression-like” pattern observed on imaging, namely decreased perfusion in areas of interest, manifesting clinically with headaches and vision impairment, may be linked to recruitment of tumor infiltrating lymphocytes into a closed cavity following infusion. In fact, a wealth of preclinical data supports the role of 9-ING-41 as an immune modulator in both in vivo and in vitro tumor models. Emerging evidence shows that GSK-3β is a central upstream regulator of major coinhibitory receptors on T cells. GSK-3β was found to regulate the transcriptional activation of programmed death-1 (PD-1) on T cells,^45^ and subsequent studies showed that pharmacological GSK-3β inhibition leads to reduction of PD-1 expression and increased function of CD8+ cytotoxic T cells in vitro and in vivo.^46,47^ Secondly, GSK-3β was also found to regulate the expression of the coinhibitory receptor Lymphocyte Activation Gene-3 (LAG-3) on CD4+ and CD8+ T cells, and other small molecule GSK-3β inhibitors were found to downregulate LAG-3 on CD4+ and CD8+ T cells and enhance tumor clearance.^48^ B16 melanoma mouse models from the Taylor lab reveal 9-ING-41 downregulates PD-1 and LAG-3 expression, leading to a synergistic effect when given sequentially or in combination with immune checkpoint inhibitors in both flank and brain melanoma models (Taylor, Personal Communication 2021).

9-ING-41 also boosts NK and effector T-cell mediated killing of tumor cells in colorectal cancer cell lines,^49^ and in both MYC-N amplified and nonamplified cell lines of neuroblastoma, exposure to 9-ING-41 leads to a boost in MHC-1 expression when stimulated with IFNy, and in MYC-N nonamplified cells a notable increase in PD-L1 expression is seen, supporting a combination approach with immune checkpoint blockade.^50^ These studies highlight the multi-pronged immune regulatory effects of GSK-3β, as well as 9-ING-41’s activity akin to an immune checkpoint inhibitor which could explain the striking pattern of recurring edema observed in some patients, requiring dose holds and ultimate extension of dosing interval to once a week.

## Conclusion

Results from the subset of 18 patients with gliomas in this FIH study demonstrate that 9-ING-41 as a single agent and in combination with lomustine is safe and well tolerated. Early evidence of clinical benefit was observed in a subset of patients, as well as pseudo-progression attributed to 9-ING-41’s known immune modulatory effects, supporting a different dosing regimen in this population. Future studies will evaluate the overall efficacy of this combination in glioblastoma patients in the temozolomide-relapse setting and explore extended treatment intervals.

## Supplementary Material

vdac012_suppl_Supplementary_Table

## References

[1] Ostrom QT , Patil N, Cioffi G, Waite K, Kruchko C, Barnholtz-Sloan JS. CBTRUS statistical report: primary brain and other central nervous system tumors diagnosed in the United States in 2013-2017. Neuro Oncol. 2020; 22(12 Suppl 2):iv1–iv96. 33123732 10.1093/neuonc/noaa200 PMC7596247

[2] Louis DN , Ohgaki H, Wiestler OD, et al. The 2007 WHO classification of tumours of the central nervous system. Acta neuropathol. 2007; 114(2):97–109. 17618441 10.1007/s00401-007-0243-4 PMC1929165

[3] Frankel SA , German WJ. Glioblastoma multiforme. J Neurosurg. 1958; 15(5):489–503. 13576192 10.3171/jns.1958.15.5.0489

[4] Pichlmeier U , Bink A, Schackert G, Stummer W; ALA Glioma Study Group. Resection and survival in glioblastoma multiforme: an RTOG recursive partitioning analysis of ALA study patients. Neuro Oncol. 2008; 10(6):1025–1034. 18667747 10.1215/15228517-2008-052 PMC2719000

[5] Wong ET , Hess KR, Gleason MJ, et al. Outcomes and prognostic factors in recurrent glioma patients enrolled onto phase II clinical trials. J Clin Oncol. 1999; 17(8):2572–2578. 10561324 10.1200/JCO.1999.17.8.2572

[6] Laperriere N , Zuraw L, Cairncross G; Cancer Care Ontario Practice Guidelines Initiative Neuro-Oncology Disease Site Group. Radiotherapy for newly diagnosed malignant glioma in adults: a systematic review. Radiother Oncol. 2002; 64(3):259–273. 12242114 10.1016/s0167-8140(02)00078-6

[7] Scott CB , Scarantino C, Urtasun R, et al. Validation and predictive power of Radiation Therapy Oncology Group (RTOG) recursive partitioning analysis classes for malignant glioma patients: a report using RTOG 90-06. Int J Radiat Oncol Biol Phys. 1998;40 (1):51–55. 9422557 10.1016/s0360-3016(97)00485-9

[8] Tsao MN , Mehta MP, Whelan TJ, et al. The American Society for Therapeutic Radiology and Oncology (ASTRO) evidence-based review of the role of radiosurgery for malignant glioma. Int J Radiat Oncol Biol Phys. 2005; 63(1):47–55. 16111571 10.1016/j.ijrobp.2005.05.024

[9] Stupp R , Mason WP, van den Bent MJ, et al.; European Organisation for Research and Treatment of Cancer Brain Tumor and Radiotherapy Groups; National Cancer Institute of Canada Clinical Trials Group. Radiotherapy plus concomitant and adjuvant temozolomide for glioblastoma. N Engl J Med. 2005; 352(10):987–996. 15758009 10.1056/NEJMoa043330

[10] Wen PY , Kesari S. Malignant gliomas in adults. N Engl J Med. 2008; 359(5):492–507. 18669428 10.1056/NEJMra0708126

[11] Stupp R , Taillibert S, Kanner AA, et al. Maintenance therapy with tumor-treating fields plus temozolomide vs temozolomide alone for glioblastoma: a randomized clinical trial. JAMA. 2015; 314(23):2535–2543. 26670971 10.1001/jama.2015.16669

[12] Stupp R , Hegi ME, Mason WP, et al.; European Organisation for Research and Treatment of Cancer Brain Tumour and Radiation Oncology Groups; National Cancer Institute of Canada Clinical Trials Group. Effects of radiotherapy with concomitant and adjuvant temozolomide versus radiotherapy alone on survival in glioblastoma in a randomised phase III study: 5-year analysis of the EORTC-NCIC trial. Lancet Oncol. 2009; 10(5):459–466. 19269895 10.1016/S1470-2045(09)70025-7

[13] Woodgett JR . Molecular cloning and expression of glycogen synthase kinase-3/factor A. EMBO J. 1990; 9(8):2431–2438. 2164470 10.1002/j.1460-2075.1990.tb07419.x PMC552268

[14] Boren J , Shryock G, Fergis A, et al. Inhibition of glycogen synthase kinase 3β blocks mesomesenchymal transition and attenuates streptococcus pneumonia-mediated pleural injury in mice. Am J Pathol. 2017; 187(11):2461–2472. 29073967 10.1016/j.ajpath.2017.07.007 PMC5809597

[15] Doble BW , Patel S, Wood GA, Kockeritz LK, Woodgett JR. Functional redundancy of GSK-3alpha and GSK-3beta in Wnt/beta-catenin signaling shown by using an allelic series of embryonic stem cell lines. Dev Cell. 2007; 12(6):957–971. 17543867 10.1016/j.devcel.2007.04.001 PMC4485918

[16] Farghaian H , Turnley AM, Sutherland C, Cole AR. Bioinformatic prediction and confirmation of beta-adducin as a novel substrate of glycogen synthase kinase 3. J Biol Chem. 2011; 286(28):25274–25283. 21606488 10.1074/jbc.M111.251629 PMC3137098

[17] Gao C , Hölscher C, Liu Y, Li L. GSK3: a key target for the development of novel treatments for type 2 diabetes mellitus and Alzheimer disease. Rev Neurosci. 2011; 23(1):1–11. 22718609 10.1515/rns.2011.061

[18] Henriksen EJ . Dysregulation of glycogen synthase kinase-3 in skeletal muscle and the etiology of insulin resistance and type 2 diabetes. Curr Diabetes Rev. 2010; 6(5):285–293. 20594161 10.2174/157339910793360888

[19] Klamer G , Song E, Ko KH, O’Brien TA, Dolnikov A. Using small molecule GSK3β inhibitors to treat inflammation. Curr Med Chem. 2010; 17(26):2873–2881. 20858169 10.2174/092986710792065090

[20] Wang H , Brown J, Gu Z, et al. Convergence of the mammalian target of rapamycin complex 1- and glycogen synthase kinase 3-β-signaling pathways regulates the innate inflammatory response. J Immunol. 2011; 186(9):5217–5226. 21422248 10.4049/jimmunol.1002513 PMC3137265

[21] An J , Yang DY, Xu QZ, et al. DNA-dependent protein kinase catalytic subunit modulates the stability of c-Myc oncoprotein. Mol Cancer. 2008; 7:32. doi:10.1186/1476-4598-7-32. PMID: 18426604; PMCID: PMC2383926. 18426604 PMC2383926

[22] Gregory MA , Qi Y, Hann SR. Phosphorylation by glycogen synthase kinase-3 controls c-myc proteolysis and subnuclear localization. J Biol Chem. 2003; 278(51):51606–51612. 14563837 10.1074/jbc.M310722200

[23] Lin SY , Xia W, Wang JC, et al. Beta-catenin, a novel prognostic marker for breast cancer: its roles in cyclin D1 expression and cancer progression. Proc Natl Acad Sci U S A. 2000; 97(8):4262–4266. 10759547 10.1073/pnas.060025397 PMC18221

[24] Pal K , Cao Y, Gaisina IN, et al. Inhibition of GSK-3 induces differentiation and impaired glucose metabolism in renal cancer. Mol Cancer Ther. 2014; 13(2):285–296. 24327518 10.1158/1535-7163.MCT-13-0681 PMC3956125

[25] Fu Y , Hu D, Qiu J, Xie X, Ye F, Lu WG. Overexpression of glycogen synthase kinase-3 in ovarian carcinoma cells with acquired paclitaxel resistance. Int J Gynecol Cancer. 2011; 21(3):439–444. 21436692 10.1097/IGC.0b013e31820d7366

[26] Liao X , Zhang L, Thrasher JB, Du J, Li B. Glycogen synthase kinase-3beta suppression eliminates tumor necrosis factor-related apoptosis-inducing ligand resistance in prostate cancer. Mol Cancer Ther. 2003; 2(11):1215–1222. 14617795

[27] Mai W , Kawakami K, Shakoori A, et al. Deregulated GSK3{beta} sustains gastrointestinal cancer cells survival by modulating human telomerase reverse transcriptase and telomerase. Clin Cancer Res. 2009; 15(22):6810–6819. 19903789 10.1158/1078-0432.CCR-09-0973

[28] Ougolkov AV , Fernandez-Zapico ME, Savoy DN, Urrutia RA, Billadeau DD. Glycogen synthase kinase-3beta participates in nuclear factor kappaB-mediated gene transcription and cell survival in pancreatic cancer cells. Cancer Res. 2005; 65(6):2076–2081. 15781615 10.1158/0008-5472.CAN-04-3642

[29] Shakoori A , Ougolkov A, Yu ZW, et al. Deregulated GSK3beta activity in colorectal cancer: its association with tumor cell survival and proliferation. Biochem Biophys Res Commun. 2005; 334(4):1365–1373. 16043125 10.1016/j.bbrc.2005.07.041

[30] Romond EH , Perez EA, Bryant J, et al. Trastuzumab plus adjuvant chemotherapy for operable HER2-positive breast cancer. N Engl J Med. 2005; 353(16):1673–1684. 16236738 10.1056/NEJMoa052122

[31] Aggarwal BB . Nuclear factor-kappaB: the enemy within. Cancer Cell. 2004; 6(3):203–208. 15380510 10.1016/j.ccr.2004.09.003

[32] Robe PA , Bentires-Alj M, Bonif M, et al. In vitro and in vivo activity of the nuclear factor-kappaB inhibitor sulfasalazine in human glioblastomas. Clin Cancer Res. 2004; 10(16):5595–5603. 15328202 10.1158/1078-0432.CCR-03-0392

[33] Kotliarova S , Pastorino S, Kovell LC, et al. Glycogen synthase kinase-3 inhibition induces glioma cell death through c-MYC, nuclear factor-kappaB, and glucose regulation. Cancer Res. 2008; 68(16):6643–6651. 18701488 10.1158/0008-5472.CAN-08-0850 PMC2585745

[34] Ugolkov A , Gaisina I, Zhang JS, et al. GSK-3 inhibition overcomes chemoresistance in human breast cancer. Cancer Lett. 2016; 380(2):384–392. 27424289 10.1016/j.canlet.2016.07.006 PMC5786372

[35] Ugolkov A , Qiang W, Bondarenko G, et al. Combination treatment with the GSK-3 inhibitor 9-ING-41 and CCNU cures orthotopic chemoresistant glioblastoma in patient-derived xenograft models. Transl Oncol. 2017; 10(4):669–678. 28672195 10.1016/j.tranon.2017.06.003 PMC5496477

[36] Ougolkov AV , Bone ND, Fernandez-Zapico ME, Kay NE, Billadeau DD. Inhibition of glycogen synthase kinase-3 activity leads to epigenetic silencing of nuclear factor kappaB target genes and induction of apoptosis in chronic lymphocytic leukemia B cells. Blood. 2007; 110(2):735–742. 17463171 10.1182/blood-2006-12-060947 PMC1924475

[37] Ougolkov AV , Fernandez-Zapico ME, Bilim VN, Smyrk TC, Chari ST, Billadeau DD. Aberrant nuclear accumulation of glycogen synthase kinase-3beta in human pancreatic cancer: association with kinase activity and tumor dedifferentiation. Clin Cancer Res. 2006; 12(17):5074–5081. 16951223 10.1158/1078-0432.CCR-06-0196 PMC2692690

[38] Hilliard TS , Gaisina IN, Muehlbauer AG, Gaisin AM, Gallier F, Burdette JE. Glycogen synthase kinase 3β inhibitors induce apoptosis in ovarian cancer cells and inhibit in-vivo tumor growth. Anticancer Drugs. 2011; 22(10):978–985. 21878813 10.1097/CAD.0b013e32834ac8fc PMC3188381

[39] Wen PY , Norden AD, Drappatz J, Quant E. Response assessment challenges in clinical trials of gliomas. Curr Oncol Rep. 2010; 12(1):68–75. 20425610 10.1007/s11912-009-0078-3

[40] Wen PY , Macdonald DR, Reardon DA, et al. Updated response assessment criteria for high-grade gliomas: response assessment in neuro-oncology working group. J Clin Oncol. 2010; 28(11):1963–1972. 20231676 10.1200/JCO.2009.26.3541

[41] Pérezleón JA , Osorio-Paz I, Francois L, Salceda R. Immunohistochemical localization of glycogen synthase and GSK3β: control of glycogen content in retina. Neurochem Res. 2013; 38(5):1063–1069. 23512644 10.1007/s11064-013-1017-0

[42] Chen W , Wang Y, Zhao B, et al. Optimal therapies for recurrent glioblastoma: a bayesian network meta-analysis. Front Oncol. 2021; 11:641878. doi:10.3389/fonc.2021.641878. PMID: 33854975; PMCID: PMC8039381. 33854975 PMC8039381

[43] Kazmi F , Soon YY, Leong YH, Koh WY, Vellayappan B. Re-irradiation for recurrent glioblastoma (GBM): a systematic review and meta-analysis. J Neurooncol. 2019; 142(1):79–90. 30523605 10.1007/s11060-018-03064-0

[44] Song J , Xue YQ, Zhao MM, Xu P. Effectiveness of lomustine and bevacizumab in progressive glioblastoma: a meta-analysis. Onco Targets Ther. 2018; 11:3435–3439. doi:10.2147/OTT.S160685. PMID: 29942135; PMCID: PMC6005326. 29942135 PMC6005326

[45] Zhao YH , Wang ZF, Pan ZY, et al. A meta-analysis of survival outcomes following reoperation in recurrent glioblastoma: time to consider the timing of reoperation. Front Neurol. 2019; 286(10):1–10. 10.3389/fneur.2019.00286 PMC6448034 30984099

[46] Taylor A , Rudd CE. Glycogen synthase kinase 3 inactivation compensates for the lack of CD28 in the priming of CD8(+) cytotoxic T-cells: implications for anti-PD-1 immunotherapy. Front Immunol. 2017; 1653(8):1–9. 10.3389/fimmu.2017.01653 PMC5732207 29312284

[47] Taylor A , Harker JA, Chanthong K, Stevenson PG, Zuniga EI, Rudd CE. Glycogen synthase kinase 3 inactivation drives T-bet-mediated downregulation of co-receptor PD-1 to enhance CD8(+) cytolytic T cell responses. Immunity. 2016; 44(2):274–286. 26885856 10.1016/j.immuni.2016.01.018 PMC4760122

[48] Taylor A , Rothstein D, Rudd CE. Small-molecule inhibition of PD-1 transcription is an effective alternative to antibody blockade in cancer therapy. Cancer Res. 2018; 78(3):706–717. 29055015 10.1158/0008-5472.CAN-17-0491

[49] Rudd CE , Chanthong K, Taylor A. Small molecule inhibition of GSK-3 specifically inhibits the transcription of inhibitory co-receptor LAG-3 for enhanced anti-tumor immunity. Cell Rep. 2020; 30(7):2075–2082.e4. 32075731 10.1016/j.celrep.2020.01.076

[50] Huntington K , Zhang S, Carneiro B, El-Deiry, W. Abstract 2676: GSK3β inhibition by small molecule 9-ING-41 decreases VEGF and other cytokines, and boosts NK and T cell-mediated killing of colorectal tumor cells. 2021; 2676–2676.

