# Supplementary material for: Malignant glioma subset from actuate 1801: Phase I/II study of 9-ING-41, GSK-3β inhibitor, monotherapy or combined with chemotherapy for refractory malignancies
Source: Neurooncol Adv. 2022 Feb 7;4(1):vdac012. doi: 10.1093/noajnl/vdac012 (PMC8989389; doi:10.1093/noajnl/vdac012)
Supplement: vdac012_suppl_Supplementary_Table [file vdac012_suppl_supplementary_table.docx]

| Part of Study | ID | Age | Gender | Histology | Molecular Data | Prior therapy | Prior Nitrosurea |
| --- | --- | --- | --- | --- | --- | --- | --- |
| 9-ING-41 + Lomustine (CCNU)  (Part 2  Combination Therapy) | 03-015 | 36 | M | WHO grade IV IDH-mutant Astrocytoma | IDH mutation,  MGMT unmethylated,  ATRX loss,  TMB low | 3 lines  (TMZ, Bev, CCNU/Bev) | Yes |
|  | 07-026 | 65 | F | Glioblastoma (Gliosarcoma) | IDH WT,  MGMT unmethylated,  TERT mutation,  EGFR amplification | 2 lines  (TMZ, BCNU) | Yes |
|  | 09-011 | 31 | M | Glioblastoma | IDH WT,  MGMT unmethylated,  TP53 mutation,  RB1 loss,  TMB low | 2 lines  (TMZ, CCNU) | Yes |
|  | 05-053 | 38 | M | Anaplastic Oligodendroglioma | IDH1 mutation,  1p/19q co-deleted, MGMT partially methylated | 2 lines  (TMZ, CCNU) | Yes |
|  | 07-030 | 52 | M | “Molecular” Glioblastoma  (Anaplastic Astrocytoma by histology) | IDH WT,  MGMT unmethylated,  EGFR amplification, TERT mutation,  ASXL1 mutation,  TMB low | 3 lines  (TMZ, TTF, BCNU) | Yes |
|  | 09-019 | 34 | M | Anaplastic (WHO grade III) IDH-mutant Astrocytoma | IDH mutation,  MGMT indeterminate,  TP53 mutation,  ATRX loss,  TMB low | 3 lines  (TMZ, CCNU/Eflornithine, Pembro/Bev) | Yes |
|  | 07-012 | 71 | F | Glioblastoma | IDH WT,  MGMT unmethylated,  TERT mutation,  PTEN loss,  EGFR amplification,  EGFR v3 mutation | 2 lines  (TMZ, CCNU) | Yes |
|  | 07-054 | 69 | M | Glioblastoma | IDH WT,  MGMT unmethylated,  TERT mutation | 2 lines  (TMZ, Ipi/Nivo/TTF) | No |
|  | 09-003 | 45 | M | Glioblastoma | IDH WT,  MGMT unmethylated,  TERT mutation,  EGFR amplification,  EGFR v3 mutation,  PTEN loss,  CDKN2A loss,  CDKN2B loss,  MTAP loss,  PIK3CA mutation | 3 lines  (TMZ, Ipi/Nivo/Bev, CCNU) | Yes |
|  | 05-080 | 46 | F | Glioblastoma | IDH WT,  MGMT unmethylated | 3 lines  (TMZ, neratinib, CCNU/TTF) | Yes |
|  | 05-079 | 55 | M | Glioblastoma | IDH WT,  MGMT unmethylated | 2 lines  (TMZ, CCNU) | Yes |
|  | 03-014 | 66 | M | G2 Astrocytoma | IDH WT,  MGMT unmethylated | 2 lines  (TMZ, CCNU/Bev) | Yes |
|  | 07-050 | 60 | M | Glioblastoma | IDH WT,  MGMT unmethylated,  TERT mutation,  EGFR amplification,  EGFR v3 mutation,  TMB low | 3 lines  (TMZ, TTF, CCNU/Bev) | Yes |
|  | 07-049 | 51 | F | Glioblastoma | IDH WT,  MGMT unmethylated,  EGFR amplification | 3 lines  (TMZ, clinical trial, TMZ) | No |
|  | | | | | | | |
| 9-ING-41 single agent  (Part 1 Monotherapy) | 09-005 | 62 | F | Glioblastoma | IDH WT,  MGMT unmethylated | 4 lines  (TMZ, TTF, Ipi/Nivo/Bev, CCNU) | Yes |
|  | 02-003 | 53 | M | Glioblastoma | IDH WT,  MGMT indeterminate,  TERT mutation,  PTEN loss | 2 lines  (TMZ, Bev) | Yes |
|  | 05-045 | 60 | F | “Molecular” Glioblastoma  (Anaplastic Astrocytoma by histology) | IDH WT,  MGMT unmethylated,  EGFR amplification,  CDKN2A loss | 1 line  (TMZ) | No |
|  | 01-023 | 52 | M | Glioblastoma | IDH WT,  MGMT unmethylated,  TERT mutation,  PTEN loss,  TP53 mutation,  NF1 mutation,  PALB2 mutation | 3 lines  (TMZ, CCNU/Bev, Carbo/Bev) | Yes |
| Patient ID in same order as swimmer plot.  Abbreviations:  Molecular: G=Grade, WT= wild type, MGMT= O^6^-methylguanine-DNA-methyltransferase, TERT= Telomerase reverse transcriptase, EGFR= Epidermal growth factor receptor, NF1= Neurofibromatosis Factor 1, RB1= retinoblastoma, TMB= Tumor Mutation Burden; ATRX= α-thalassemia X-linked mental retardation  Therapy: TMZ= temozolomide, Bev= Bevacizumab, CCNU= lomustine, BCNU= carmustine, TTF= tumor-treating fields/Optune, Pembro= Pembrolizumab, Ipi= Ipilimumab, Nivo=Nivolumab, Carbo=Carboplatin | | | | | | | |
